# Glia in the cytokine-mediated onset of depression: fine tuning the immune response

**DOI:** 10.3389/fncel.2015.00268

**Published:** 2015-07-10

**Authors:** Wendy K. Jo, Yuanyuan Zhang, Hinderk M. Emrich, Detlef E. Dietrich

**Affiliations:** ^1^Research Center for Emerging Infections and Zoonoses (RIZ), University of Veterinary Medicine HannoverHannover, Germany; ^2^Clinic for Mental Health, Hannover Medical SchoolHannover, Germany; ^3^Burghof-KlinikRinteln, Germany

**Keywords:** depression, glia, inflammation, KYN pathway, vitamin D, VDR

## Abstract

Major depressive disorder (MDD) is a mood disorder of multifactorial origin affecting millions of people worldwide. The alarming estimated rates of prevalence and relapse make it a global public health concern. Moreover, the current setback of available antidepressants in the clinical setting is discouraging. Therefore, efforts to eradicate depression should be directed towards understanding the pathomechanisms involved in the hope of finding cost-effective treatment alternatives. The pathophysiology of MDD comprises the breakdown of different pathways, including the hypothalamus-pituitary-adrenal (HPA) axis, the glutamatergic system, and monoaminergic neurotransmission, affecting cognition and emotional behavior. Inflammatory cytokines have been postulated to be the possible link and culprit in the disruption of these systems. In addition, evidence from different studies suggests that impairment of glial functions appears to be a major contributor as well. Thus, the intricate role between glia, namely microglia and astrocytes, and the central nervous system’s (CNSs) immune response is briefly discussed, highlighting the kynurenine pathway as a pivotal player. Moreover, evaluations of different treatment strategies targeting the inflammatory response are considered. The immuno-modulatory properties of vitamin D receptor (VDR) suggest that vitamin D is an attractive and plausible candidate in spite of controversial findings. Further research investigating the role of VDR in mood disorders is warranted.

## Introduction

Depression is a common neuropsychiatric disorder constituting one of the leading causes of disability worldwide, estimated to affect 350 million people, and projected to be the leading cause of disease burden by 2030 (World-Health-Organization, 2008; World-Federation-for-Mental-Health, 2012). The comorbid state of depression together with a number of other physical and psychiatric disorders results in increased disability and mortality rates. Among some of the most frequently associated disorders are cardiovascular disease, cancer, diabetes, obesity, attention deficit hyperactivity disorder, and Alzheimer’s disease (Schillani et al., 2012; Hannon et al., 2013; Mavrides and Nemeroff, 2013; Wuwongse et al., 2013; Di Trani et al., 2014).

Although major depressive disorder (MDD) has been extensively studied, rates of depression have not decreased over the past decades. According to a review comparing cases of mental disorders between 2004 and 2010 in the European Union, rates in this period remained more or less stable with the increase in better detection and health care systems. However, in a previous study the prevalence of persons affected each year was estimated to be 38.2%, depression being the second most prevalent form of mental disorders (6.9%; Wittchen et al., 2011). Moreover, with the currently prescribed pharmacological agents, relapse rates are as high as 50%, only one-third of patients achieving complete remission. Therefore, there is a clear necessity to increase the amount of research investigating the molecular mechanisms pertaining depression. The aim of this review is to connect the different pathophysiological pathways of depression in an effort to find suitable treatments.

## Major Depressive Disorder

MDD along with bipolar disorder are categorized under mood disorders, also referred to as affective disorders. MDD is a condition characterized by one or more depressive episodes along with pathophysiological changes in the brain (Muller and Schwarz, 2007). These depressive episodes appear without a history of manic or hypomanic episodes, as in the case of bipolar disorder. Typical symptoms include low mood, anhedonia, appetite alterations, sleep disturbances, fatigue, poor concentration, and feelings of worthlessness, among others (World-Federation-for-Mental-Health, 2012). Moreover, MDD is attributed as a risk factor for suicide attempts and drug abuse (Davis et al., 2008). Furthermore, depressive episodes are often recurrent, incrementing individual and social burden (World-Federation-for-Mental-Health, 2012).

Characteristic pathophysiological hallmark features include monoamine depletion, glucocorticoid receptor (GR) resistance, and excess of glutamate, corticotrophin-releasing hormone and cortisol levels, therefore compromising the monoaminergic and glutamatergic neurotransmission along with the hypothalamus-pituitary-adrenal (HPA) axis activity (Muller and Schwarz, 2007).

The onset of MDD is complex and can be triggered by several different factors. While genetic factors have been estimated to contribute 30–40% of the etiology of MDD, the remaining factors are accounted for by environmental risk factors. Negative traumatic events during lifetime (e.g., child abuse, parental divorce, death of a loved one, etc.) can lead to MDD. However, this depends on the genetic susceptibility, gender, family history, and personality traits of the person affected (Heim and Binder, 2012). Family risk factors are estimated to increase its incidence two to three times (Wilde et al., 2014). Development of MDD is suggested to be the outcome of the interplay between both genetic and environmental risk factors rather than the result of one factor. For instance, it was demonstrated in one study that interactions between experiences of childhood abuse and genetic variations of both serotonin transporter and corticotrophin receptor 1 genes lead to the development of MDD (Ressler et al., 2010).

The trend towards the study of MDD from an epigenetic approach is increasing with the aid of new available technologies. These studies allude to environmental stressors that can modify epigenetically the expressions of susceptible genes, thereby resulting in the dysfunction of relevant mechanisms in MDD (Mill and Petronis, 2007; Fass et al., 2014). For example, histone acetylation and methylation were reported in animal models of depression (Tsankova et al., 2006; Fuchikami et al., 2009). Similar mechanisms were proposed to occur in depressed patients as evidenced by post-mortem brain and serological studies (Cruceanu et al., 2013; Sun et al., 2013). Moreover, exposure to early negative experiences was shown to alter methylation of GR and brain derived neurotropic factor (BDNF; Elliott et al., 2010; Perroud et al., 2013). Furthermore, epigenetic modifications in the gene promoter region of BDNF in mood disorder patients have been shown by several studies to affect the patient’s response to the treatment received (D’Addario et al., 2012; Lopez et al., 2013; Tadić et al., 2014). These studies suggest the evaluation of epigenetic changes in BDNF as a potent biomarker for treatment response in mood disorder patients.

## Crosstalk Between the Monoaminergic and Glutamatergic Systems

Over the past half-century, comprehensive studies concentrating on the monoamine hypothesis have led to the development of a number of treatments, whose target is to increase the availability of monoamines, such as serotonin and norepinephrine (Charney, 1998). Depletion of serotonin is commonly attributed to the shift from the serotonin pathway to the kynurenine (KYN) pathway in the catabolism of the essential amino acid tryptophan. The KYN pathway can be mediated by a variety of cell types in the body. However, the resulting downstream product depends on the kind of enzymes that each cell type has for the processing of tryptophan. In the central nervous system (CNS), the KYN pathway appears to be mainly mediated by astrocytes, microglia, and infiltrating macrophages. It is initiated by activation of indoleamine 2, 3-dioxygenase (IDO) catabolizing tryptophan into KYN, which can be further converted to kynurenic acid (KA) or quinolinic acid (QUIN). KA and QUIN have contrasting roles influencing the glutamatergic system, the first acting as antagonist and the latter as agonist of the glutamate N-methyl-D-aspartate receptor (NMDAr; Campbell et al., 2014). Microglia are the main producers of QUIN in the brain, whereas astrocytes are the CNS-key cells involved in KA synthesis. This is explained by the fact that microglia express kynurenine 3-monooxygenase (KMO), the rate-limiting enzyme in the production of QUIN. Conversely, astrocytes exclusively express kynurenine aminotransferases, which are essential in the conversion of KYN to KA (Guillemin et al., 2001, 2005). IDO, the initiator of the KYN pathway, has been reported to be induced by a number of different pro-inflammatory cytokines, such as interferon-γ (IFN-γ), tumor necrosis factor α (TNF-α), and interleukin-6 (IL-6). Therefore, the activation of the KYN pathway is attributed to be cytokine-mediated (O’Connor et al., 2009a; Kim et al., 2012; Campbell et al., 2014).

MDD also compromises the glutamatergic system characterized by the increased activation of NMDAr (Trullas and Skolnick, 1990). QUIN and glutamate are agonists of NMDAr, levels of both being found to be elevated in MDD. Inflammation can induce the synthesis of QUIN through the activation of the KYN pathway in microglia and macrophages in the CNS (Guillemin et al., 2003). QUIN acts not only as an agonist of NMDAr at the glycine-binding site, but has also been shown to stimulate the release of glutamate from neurons, inhibiting its re-uptake by astrocytes (Tavares et al., 2002, 2005). Furthermore, several studies indicate that QUIN toxicity can also lead to lipid peroxidation and nitrosative stress (Behan et al., 1999; Aguilera et al., 2007). On the other hand, KA is an antagonist of the NMDAr with antioxidant activity found to induce neuro-protective effects in ischemic and oxidative stress rodent models (Nozaki and Beal, 1992; Lugo-Huitrón et al., 2011). Nevertheless, the role of KA in relation to depression is not yet clear. A recent longitudinal study of suicide attempters with depression showed that cerebrospinal fluid (CSF) QUIN levels are increased, while CSF KA levels are decreased over a 2-year period along with an increase in IL-6 and worsening of depressive and suicidal symptoms (Bay-Richter et al., 2015). However, treatment of the pro-inflammatory cytokine IFN-α in patients with hepatitis C virus (HCV) was shown to elevate both QUIN and KA concentrations in the CSF, with only QUIN levels found to be correlated to depression scores (Raison et al., 2010). IFN-α therapy has been widely used to treat HCV, though it is reported to induce depressive and manic symptoms in 30–50% of patients (Bonaccorso et al., 2002; Constant et al., 2005). Moreover, studies evaluating the KYN pathway in the periphery have reported an increased KYN/KA ratio associated with depressive symptoms in individuals with HCV treated with IFN-α, and a reduced KA/QUIN ratio in MDD patients compared with controls (Wichers et al., 2005; Savitz et al., 2015). Of note, new findings using skeletal muscle-specific peroxisome proliferator-activated receptor gamma coactivator 1-alpha1 (PGC-1α1) transgenic mouse model suggest that physical exercise is beneficial in stress-induced depression due to its modulation of the KYN pathway. Enhanced PGC-1α1 in the skeletal muscle stimulated KA synthesis, thus reducing peripheral KYN, which, contrastingly to KA, can readily cross the BBB (Agudelo et al., 2014). Interestingly, contrary to the observations in depression, elevated levels of KA have been associated with the cognitive deficits in schizophrenic patients (Erhardt et al., 2004; Linderholm et al., 2012). Augmented inhibition of the NMDAr by KA appears to cause changes in the glutamatergic system affecting the dopaminergic neurotransmission, a hallmark feature of schizophrenia (Wu et al., 2007; Pocivavsek et al., 2011).

## Glial Pathology in Relation to Neuro-Pathophysiological Alterations

Accumulating evidence suggests that glial pathology is a prominent feature in MDD. Glial cells are the non-neuronal cells in the CNS, comprised of astrocytes, microglia and oligodendrocytes. Human post-mortem studies of mood disorder patients consistently demonstrate a significant decrease in the number of glia in the pre-frontal cortex and limbic structures of the brain (Rajkowska et al., 1999; Cotter et al., 2001; Bowley et al., 2002; Hamidi et al., 2004; Altshuler et al., 2010). Moreover, it was reported that glial ablation in the pre-frontal cortex of mice induces anhedonia, anxiety and helplessness behavior after exposure to chronic unpredictable stress procedures. The depressive-like behaviors generated were assessed by sucrose preference test, novelty suppressed feeding test, forced swim test, and two-way active avoidance test (Banasr and Duman, 2008). Furthermore, it was shown in another study by the same group that exposure to chronic unpredictable stress can likewise induce glial dysfunction resulting in depressive-like behavior (Banasr et al., 2010). It was proposed that initial glial impairment by different stressors leads to neuronal damage in the progression of the disorder (Rajkowska and Miguel-Hidalgo, 2007).

Astrocytes have multifaceted roles in the CNS, participating actively in synaptic information transmission, secretion of synaptogenic molecules, such as BDNF, modulation of the BBB, mediation of the immune system, and uptake of cytotoxic molecules from the extracellular space, such as glutamate (Jo et al., 2014). Astrocytes express the excitatory amino acid transporters 1 and 2 (EAAT1 and EAAT2), which enables them to transport glutamate to the intracellular compartment. Once inside, glutamate is further converted to glutamine by the enzyme glutamine synthase (Anderson and Swanson, 2000). Astrocytes also take part in the KYN pathway synthesizing KA but not QUIN (Guillemin et al., 2001). Accumulating data from human post-mortem studies of depressed patients reveal a significant decrease in the number of astroglial cells and less coverage of blood vessels by astrocytic end-feet (Johnston-Wilson et al., 2000; Gittins and Harrison, 2011; Rajkowska et al., 2013). Loss of astrocytes has been demonstrated by studies reporting decreases of the astrocytic marker glial fibrillary acidic protein and both enzymes EAAT1 and EAAT2 (Johnston-Wilson et al., 2000; Miguel-Hidalgo et al., 2010; Gittins and Harrison, 2011). Moreover, morphometric analyses in the anterior cingulate cortex region of depressed suicides revealed the presence of hypertrophic astrocytes, indicating possible astrocytic activation in the area (Torres-Platas et al., 2011). Furthermore, significantly increased genome-wide DNA methylation patterns were reported in the pre-frontal cortex of depressed patients (Nagy et al., 2014). This was found alongside the down-regulation of astrocytic markers in the same study, implying possible epigenetic regulations associated with astrocytic pathology (Nagy et al., 2014). The evidence reported suggests that in the event of the activation of the KYN pathway by pro-inflammatory cytokines, without functional astrocytes, QUIN/KA ratio would be increased. Moreover, glutamate clearance from the extracellular space would also result disturbed. Hence, elevated levels of QUIN and glutamate would promote NMDAr agonism disrupting the glutamatergic system.

Microglia are professional phagocytes regarded as CNS-resident immune cells. They are the primary sentinels patrolling the CNS, alert to any potential harmful event including pathogen invasion. Moreover, their role is also extended to debris clearance, trophic support to neurons, and synaptic pruning during neurogenesis (Ousman and Kubes, 2012). In human post-mortem studies, higher microglial activation was observed in different regions including anterior cingulate cortex, pre-frontal cortex and hippocampus of depressed suicides (Steiner et al., 2008; Torres-Platas et al., 2014). QUIN levels were also found to be dysregulated in different regions of the brain. Elevated microglial QUIN expression was reported in the cingulate cortex, whereas a decrease or no change in microglial QUIN immunoreactivity was observed in the hippocampus of acutely depressed patients (Steiner et al., 2011; Busse et al., 2014). Supporting this notion, it was shown in parallel that chronic unpredictable stress in mice led to depressive-like behavior such as decrease in sucrose consumption and social exploration along with microglial proliferation and activation, ending in subsequent microglial apoptosis (Kreisel et al., 2014). However, blockade of microglial initial activation minimized the aforementioned detrimental effects (Kreisel et al., 2014). Moreover, microglial cell culture experiments showed that adding IFN-α induced microglia activation and production of pro-inflammatory cytokines, such as IL-1β, IL-6 and TNF-α (Zheng et al., 2015). Furthermore, in another study, IFN-α treated microglia stimulated the expression of inducible nitric oxide synthase (iNOS) and secretion of NO, alongside the down-regulation of heme oxygenase-1 (HO-1), a potent anti-inflammatory and neuro-protective protein (Lu et al., 2013). Hence, it was concluded that IFN-α may contribute to the pathogenesis of depression by triggering inflammation, oxidative stress, and abrogating anti-inflammatory and neuro-protective responses (Lu et al., 2013; Zheng et al., 2015). It is therefore suggested that with the activation of microglia, the secreted pro-inflammatory cytokines would activate the KYN pathway. Tryptophan degradation is then shifted to the KYN pathway, resulting in depletion of monoamines and production of QUIN, compromising the monoaminergic and glutamatergic systems.

Oligodendrocytes are the myelin-cell producers in the CNS enveloping neuronal axons, allowing communication through action potentials. Among the glia, oligodendrocytes are particularly vulnerable to stress-related insults (Edgar and Sibille, 2012). Oligodendrocytic dysfucntion in mood disorders is evidenced by human post-mortem studies reporting reduced numbers of oligodendrocytes in the pre-frontal cortex and amygdala (Hamidi et al., 2004; Uranova et al., 2004). Perineuronal oligodendrocytes have been identified in later studies as the subtype of oligodendrocytic cell affected. Perineuronal oligodendrocytes are non-myelin producer cells that undergo functional changes following an injury allowing them to synthesize myelin and possibly rescue neuronal axons from de-myelination. The loss of perineuronal oligodendrocytes was reported in the pre-frontal cortex of mood disorders and schizophrenia (Vostrikov et al., 2007; Szuchet et al., 2011). Moreover, this decrease in the number of perineuronal oligodendrocytes was correlated to cytoarchitectural abnormalities in genome-wide association analyses (Kim and Webster, 2010).

Despite the differences discussed regarding the type of glia affected and its state (resting or activated) in the different neuropsychiatric disorders, the evidence presented so far suggests that dysfunctional glia play an active role in the abrogation of the monoaminergic and glutamatergic neurotransmission systems, contributing to cognitive deficits and behavioral changes in mood disorders.

## Depression as an Inflammatory Disease

The inflammatory hypothesis of depression has gained momentum since it first appeared two decades ago, proposing MDD as a cytokine-mediated disorder (Maes et al., 1995; Raison et al., 2006; Dantzer et al., 2008). An increasing amount of clinical evidence has shown the presence of augmented levels of pro-inflammatory markers in the periphery and in the CNS (Maes et al., 1993; Rotter et al., 2013; Dahl et al., 2014). Moreover, high rates of comorbidity have been reported between depression and immune-associated disorders, such as cardiovascular disease, multiple sclerosis, and autoimmune disorders (Maes et al., 1993; Bachen et al., 2009; Byatt et al., 2011). Meta-analyses of studies associating inflammatory markers with MDD reported significantly higher levels of TNF-α, IL-6, and C-reactive protein (CRP) in depressed patients (Dowlati et al., 2010; Valkanova et al., 2013). A recent longitudinal study conducted in England revealed that children with elevated serum IL-6 and CRP levels were more at risk to develop depression and psychosis later in life (Khandaker et al., 2014). Furthermore, several studies report that cytokine therapy, such as IFN-α, can generate depressive and manic symptoms in HCV and cancer patients (Bonaccorso et al., 2002; Capuron et al., 2002; Constant et al., 2005). In one study, serum levels of TNF-α, IL-6 and soluble IL-2 receptor were shown to be upregulated after IFN-α treatment (Wichers et al., 2007). In another study, elevated levels of QUIN and KA were found in the CSF of patients treated with peripheral administration of IFN-α (Raison et al., 2010). In further support of the inflammatory hypothesis of depression, studies using animal models have demonstrated that administering lipopolysaccharides (LPS) as well as pro-inflammatory cytokines induce depressive-like behaviors such as anhedonia, assessed by sucrose/saccharine preference tests (Yirmiya, 1996; De La Garza, 2005).

It is important to point out that despite this overwhelming evidence of an association between inflammation and depression, failed attempts to prove this correlation have also been reported (Haack et al., 1999; Steptoe et al., 2003). Moreover, the profile of cytokines in suicidal and non-suicidal patients is distinct (Kim et al., 2008). Cytokine level changes in response to antidepressant treatment are also varied (Hannestad et al., 2011). The heterogeneous results indicate that MDD is a rather complex disorder with multiple variants involving the inflammatory response participation to a great extent, but not in all cases. Nevertheless, cytokines can mediate several pathways which are crucial in the pathophysiology of depression. As discussed earlier, pro-inflammatory cytokines can induce IDO, therefore activating the KYN pathway. This event can lead to the dysregulation of the monoaminergic and glutamatergic neurotransmissions. Moreover, the GR resistance has been attributed to be cytokine mediated. A number of cytokines, such as IL-1α, TNF-α and IFN-α, have been reported to inhibit GR function (Wang et al., 2004; Hu et al., 2009; Van Bogaert et al., 2011). Without competent GRs, glucocorticoids such as cortisol cannot exert its anti-inflammatory effects, thus ending in the exacerbation of the inflammatory response (Pace et al., 2007). In addition, GR resistance promotes hyperactivation of the HPA axis leading to the overproduction of glucocorticoids, hallmark features of MDD (Anacker et al., 2011). On the other hand, enhanced activation of the immune system can also be mediated by inadequate levels of glucocorticoids, resulting in the development of depressive symptoms (Raison and Miller, 2003). For example, in the case of atypical depression, corticotrophin-releasing hormone deficiency, hypoactivity of the HPA axis, and increased inflammatory response are often found in these patients (Gold and Chrousos, 2002). It is proposed that insufficient glucocorticoid signaling, either by low levels of cortisol or GR resistance, can lead to a cytokine-mediated onset of depression (Raison and Miller, 2003).

Different hypotheses have been postulated in order to explain the chronic low-grade inflammatory status in mood disorders. A number of lifestyle factors have been proposed to affect the immune status in depressed individuals, such as non-healthy dietary habits (e.g., high saturated fats, processed meats, refined carbohydrates, etc.), physical inactivity, smoking habits, and vitamin D deficiency (Berk et al., 2013). Chronic psychological stress is a well-studied risk factor suggested to induce GR resistance and trigger the inflammatory cascade (Miller et al., 2002; Raison et al., 2006; Cohen et al., 2012). From the genetic perspective, polymorphisms in the genes *PSMB4* (proteasome β4 subunit) and *TBX21* (T bet) that result in T-cell dysfunction, were reported to contribute to the pathology of MDD (Wong et al., 2008; Berk et al., 2013). A recent meta-analysis of 28 studies identified significant associations between depression and infections in Borna disease virus (BDV), herpes simplex virus-1, varicella zoster virus, Epstein-Bar virus (EBV), and *Chlamydophila trachomatis*. Results indicated that patients with depression are 3.25 times more likely to be infected with BDV (Wang et al., 2014). However, negative findings have also been reported (Bennett et al., 2012; Hornig et al., 2012; Pearce et al., 2012). Despite controversies aroused concerning direct virus association and mood disorders, several studies demonstrated that there is a higher risk of developing mood disorders later in life among individuals who have had a severe infection (Goodwin, 2011; Benros et al., 2013). In accordance with these findings, it was previously suggested that stress could alter the immune system, thereby resulting in reactivation of persistent viruses in the CNS, further enhancing the inflammatory reaction (Dietrich et al., 1998). Experimental evidence demonstrated that rats exposed to stress developed intestinal permeability and bacterial translocation. LPS from bacterial translocation activated toll-like receptor 4 (TLR4) in the brain triggering the neuroinflammatory response (Garate et al., 2011).

The systemic inflammatory response generates circulating cytokines and other mediators that can gain access to the brain through different pathways. The mechanisms involve cytokine signal propagation, activation of vagal afferents, active transport across the BBB, and diffusion at sites where the BBB is leaky or absent. It is important to indicate that this communication is bi-directional (Maier, 2003). Response within the CNS is consequently triggered. Microglia and astroglia, important immune-regulators, become activated and further enhance the inflammatory response (Biesmans et al., 2013). In addition, pro-inflammatory cytokines can activate the KYN pathway in both glial cell types resulting in the dysregulation of the monoaminergic and glutamatergic systems. On account of the evidence linking inflammation and depression it was proposed that depression is a byproduct of the immune systems in its attempt to fight infection (Raison and Miller, 2013).

## Therapeutic Intervention

Among the many types of antidepressants that exist to date, tricyclic antidepressants, monoamine oxidase inhibitors, selective serotonin re-uptake inhibitors (SSRIs), and serotonin-norepinephrine re-uptake inhibitors are some of the most common types to be prescribed (Anderson et al., 2012; Klomp et al., 2014). Tricyclic antidepressants were the most widely prescribed pharmacological agent. However, its supplementation was found to lead to severer and lethal (overdoses) side effects compared with the other types of antidepressants (Boyce and Judd, 1999). Administering monoamine oxidase inhibitors was found to carry dangerous side effects as well, and these inhibitors are now used mainly in treatment-resistant depression (Finberg, 2014). SSRIs have become the first choice of antidepressants over the last two decades. Although their efficacy is comparable to the tricyclic antidepressants, the preferred choice for SSRIs arises from the fact that they are safer, have better tolerability, and lower rates of treatment discontinuation (Anderson, 2000). However, response rates are about 50%, full remission is achieved only in one-third of patients responding to treatment, and relapse is more often than not the case (Trivedi et al., 2006). Therefore, the current drawbacks of presently available antidepressants in a clinical setting lead to the search for better cost-effective treatments and the consideration of alternative therapies. For instance, the triple reuptake inhibitors, which tackle the monoaminergic system by inhibiting serotonin, norepinephrine, and dopamine transporters appear to be a promising antidepressant agent (Chen and Skolnick, 2007). However, their efficacy and side-effects are currently under investigation (Tran et al., 2012; Risinger et al., 2014). The use of the NMDAr antagonist ketamine as an antidepressant has gained some validity in the last years. Despite its status as a popular abusive drug causing hallucinations and psychosis, its use in treatment-resistant depressive patients by targeting the glutamatergic system has shown faster and greater improvements (Zarate et al., 2006; Murrough et al., 2013). However, safety and effectiveness of ketamine usage in the long term needs to be further evaluated (Rush, 2013).

## Targeting Inflammation in Depression

On account of increasing evidence pointing towards the consideration of MDD as a cytokine-mediated disorder, a number of studies have investigated the implication of current available agents on the immune response. The outcome suggests that pharmacological antidepressants can exert anti-inflammatory effects as a mechanism of action. Studies assessing fluoxetine and the tricyclic antidepressants clomipramine and imipramine demonstrated that these agents are capable of modulating the immune response by inhibiting the activation of glial cells in cell culture models (Hwang et al., 2008; Obuchowicz et al., 2014). Moreover, although the meta-analysis of 22 studies indicated that SSRIs appeared to decrease IL-6 and TNF-α cytokine levels to some extent, it also demonstrated that despite the reduced depressive symptoms found in the studies, an overall effect in the decline of IL-6 and TNF-α after treatment was not found. Noteworthy, IL-1β levels were found to be significantly reduced (Hannestad et al., 2011).

Other treatment alternatives aiming to target the chronic low-grade inflammatory response present in a subgroup of depressive patients have also been sought. These include non-steroidal anti-inflammatory drugs (NSAIDs), minocycline, omega-3 fatty acids, and vitamin D, among others. Usage of selective cyclooxygenase (COX)-2 and non-selective COX inhibitors NSAIDs, namely celecoxib and aspirin, to treat MDD has yielded varied results. A recent meta-analysis of 11 studies evaluating the use of selective COX-2 or non-selective COX inhibitors, including seven randomized clinical trials (RCTs) and four cohort studies, found no overall significant changes in the response to either type of NSAIDs (Eyre et al., 2015). However, in the studies evaluated, effectiveness to reduce depressive symptoms had better outcomes when celecoxib was used as an add-on therapy (Muller et al., 2006; Akhondzadeh et al., 2009). On the other hand, the usage of the tetracycline derivative minocycline to treat mood disorders is currently undergoing clinical trials (Savitz et al., 2012; Dean et al., 2014). Minocycline has been suggested to regulate neuroplasticity by exerting anti-apoptotic, anti-inflammatory and anti-oxidative properties (Soczynska et al., 2012). Moreover, it has been shown to modulate the glutamatergic and monoaminergic systems (O’Connor et al., 2009b; Wixey et al., 2011). Minocycline has been demonstrated to inhibit activation and proliferation of microglia, therefore regulating the immune response (Tikka et al., 2001; Henry et al., 2008). Furthermore, minocycline has been also shown to facilitate the recovery of sickness behavior and down-regulate pro-inflammatory cytokines in mice treated with either LPS or TNF-α (Henry et al., 2008; Zheng et al., 2015).

In regards to omega-3 fatty acids, although their supplementation has been reported to exert beneficial effects in mood disorders (presumably due to its anti-inflammatory properties), a recent meta-analysis of 13 separate RCTs demonstrated no significant effect on MDD (Nemets et al., 2002; Su et al., 2003; Bloch and Hannestad, 2012).

In the case of vitamin D, a meta-analysis of 14 observational studies falling in the category of case-control, cross-sectional and cohort demonstrates that low levels of vitamin D are significantly associated with depression (Anglin et al., 2013). However, the effectiveness of its supplementation in reducing depressive symptoms remains controversial (Jaddou et al., 2012; Kjaergaard et al., 2012). In a recent meta-analysis of seven RCTs, it was shown that supplementation of vitamin D had no overall effect on depressive symptoms. Interestingly, the meta-analysis also points out that better outcomes were achieved with vitamin D supplementation only in cases where clinically significant depressed patients participated in the study. In addition, the largest effect was achieved when vitamin D was administered as an adjuvant (Shaffer et al., 2014). It is noteworthy to mention that although vitamin D intake is broadly considered to induce no adverse effects, overdoses of vitamin D have proven to cause hypercalcemia (Hathcock et al., 2007). Moreover, a study conducted in older community-dwelling women reported that annual high doses of vitamin D (500,000 IU) in a period of 3–5 years increased the risk of falls and fractures, contrary to what was expected (Sanders et al., 2010).

## Vitamin D—A Potential Therapeutic Agent?

Vitamin D is regarded as the sunshine vitamin due to the fact that the main natural source of vitamin D is UV-mediated in the epidermal layer of the skin through sunlight stimulation. UV-light converts 7-dehydrocholesterol to pre-vitamin D_3_ form, which is later hydroxylated to the major circulating form vitamin D_3_ by the enzyme vitamin D 25-hydroxylase. A final hydroxylation is conducted by 1-α-hydroxylase generating the bioactive form 1,25(OH)_2_D_3_, also known as calcitriol. However, other natural sources include cod-liver oil, oily fish, butter, cream, and egg yolk (Baeke et al., 2010). This light-dependent source and the fact that vitamin D was demonstrated to act on the brain, spinal cord and several endocrine tissues led to suggest a linkage between vitamin D and neuropsychiatric disorders in individuals less exposed to sunlight (Stumpf and Privette, 1989). Evidence of vitamin D deficiency was later shown in seasonal affective disorder patients (Lansdowne and Provost, 1998). Since then, a number of studies on vitamin D and its effect on MDD have been conducted, yielding highly controversial results, as discussed above. Moreover, vitamin D deficiencies are also reported in the general population (Holick, 2006). Nevertheless, the importance of contemplating vitamin D as a plausible alternative to treat MDD arises not only from its cost-effectiveness and low adverse effects, but is also due to its influence in the immune response.

Vitamin D is a steroid hormone with pleiotropic effects. Aside from its long-recognized role in regulating calcium and phosphorous balance, vitamin D can also influence cell differentiation and proliferation, as well as modulate the immune system. In the CNS, vitamin D can act as an immune-regulator, and as stimulator of neurotrophic factors and neurotransmitters expression (Di Rosa et al., 2011; Eyles et al., 2011; Gezen-Ak et al., 2011). Vitamin D has two main receptors, membrane-associated rapid response steroid-binding (MARRS) and vitamin D receptor (VDR), which are critical for its different regulatory properties (Khanal and Nemere, 2007; Meyer et al., 2010). MARRS is a membrane receptor that when bound to vitamin D induces rapid non-genomic responses, such as modulation of calcium concentrations and activity of protein kinase C (Khanal and Nemere, 2007). VDR, on the other hand, is a transcription factor that regulates the expression of multiple genes and is responsible for the non-classical responses of vitamin D (Meyer et al., 2010). Upon binding to vitamin D, VDR translocates to the nucleus and heterodimerizes with the retinoid X receptor (RXR). Subsequently, the VDR-RXR complex formed binds to vitamin D responsive elements in the DNA to activate or repress the expression of vitamin D target genes (Fetahu et al., 2014). Moreover, the VDR gene has large cytosine/guanine dinucleotide (CpG) repeats at the promoter region that are susceptible to epigenetic modifications. Conversely, VDR can also modulate the epigenome, inducing DNA methylation and chromatin modulation (Fetahu et al., 2014).

VDR is expressed in more than 38 types of cells, including immune cells (e.g., monocytes, dendritic cells, activated B and T cells) and CNS cells (e.g., neurons, astrocytes, and microglia; Di Rosa et al., 2011; Cui et al., 2013; Smolders et al., 2013). VDR activation was reported to regulate the innate immune response by inducing tolerogenic dendritic cells, inhibiting type 1 T helper (Th1) cell responses, as well as downregulating TLR2, TLR4, and TLR9, inducing decreased expressions of IL-6 (Dickie et al., 2010). Studies with neurons also showed that VDR can regulate the expressions of nerve growth factor and iNOS (Gezen-Ak et al., 2011; Dursun et al., 2013). In addition, the VDR gene has been reported to be associated with viral and bacterial infections. In one study, Epstein-Barr virus nuclear antigen 3 (EBNA-3; produced by EBV) was demonstrated to bind to VDR inducing blockage of VDR-dependent genes, thus protecting cells from VDR-induced growth arrest and/or apoptosis (Yenamandra et al., 2010). Another study showed downregulation of VDR expression in monocytes by *Borrelia burgdorferi* infection (Salazar et al., 2009). In addition, human immunodeficiency virus (HIV) was proved to downregulate VDR expression by inducing its hypermethylation in T cells. This event resulted in the activation of the renin angiotensin system and generation of reactive oxidative species, consequently leading to T cell apoptosis (Chandel et al., 2013). Moreover, vitamin D elicits the expression of the anti-microbial peptides cathelicidin and defensin, important for counteracting infection (Gombart, 2009).

Despite the substantial amount of evidence linking VDR and the immune response, its role in the regulation of the inflammatory response in mood disorders remains to be elucidated. Only two studies to date have reported an association between VDR gene variant and susceptibility to develop depressive symptoms in old age (Kuningas et al., 2009; Glocke et al., 2013). Moreover, studies evaluating the relationship of vitamin D and the cytokine network in mood disorders fall short of expectations. In a recent study, it was reported that suicide attempters had significantly lower levels of vitamin D and higher levels of the pro-inflammatory cytokine IL-1β circulating the blood in comparison to non-suicidal depressed participants and healthy controls. In another study evaluating obese women with polycystic ovary syndrome, vitamin D deficiency was found to be associated with higher depressive symptoms and higher CRP, independent of polycystic ovary syndrome presence (Moran et al., 2015). Furthermore, studies regarding vitamin D immuno-modulatory effects on microglia and astrocytes in depression models are limited in spite of the considerable amount of evidence showing the relevance of these glial cells in the mediation of the CNS immune system. Microarray analysis of primary mixed CNS glia cultures showed that when challenged by a mixed group of Th1 or Th2 cytokines (pro-inflammatory and anti-inflammatory cytokines, respectively) the dopaminergic receptor and enzymes involved in vitamin D metabolism were affected. The expression of 25-hydroxylase (CPY27B1), which generates calcitriol, became upregulated when stimulated with pro-inflammatory cytokines of the Th1 group, whereas the expression of 24-hydroxylase (CPY24A1), the enzyme that catabolizes calcitriol, became downregulated when stimulated with anti-inflammatory cytokines of the Th2 group (Lisak et al., 2009). In line with these results are findings from another study, in which the addition of IFN-γ and TNF to primary human microglia and astrocytic cultures showed upregulation of CYP27B1 mRNA expression, which was reduced in the presence of active vitamin D (Smolders et al., 2013). In a recent report, vitamin D deficient microglia cultures stimulated by TLR agonists showed a decrease of TNF-α and IL-6. Moreover, lower phagocytosis and intracellular killing rates of *Escherichia coli* were also observed (Djukic et al., 2014). In an Alzheimer’s disease model, human primary microglia triggered by β-amyloid appear to influence the expression of various inflammatory-related proteins, as well as the upregulation of IDO and VDR (Walker et al., 2006).

**Figure 1:**
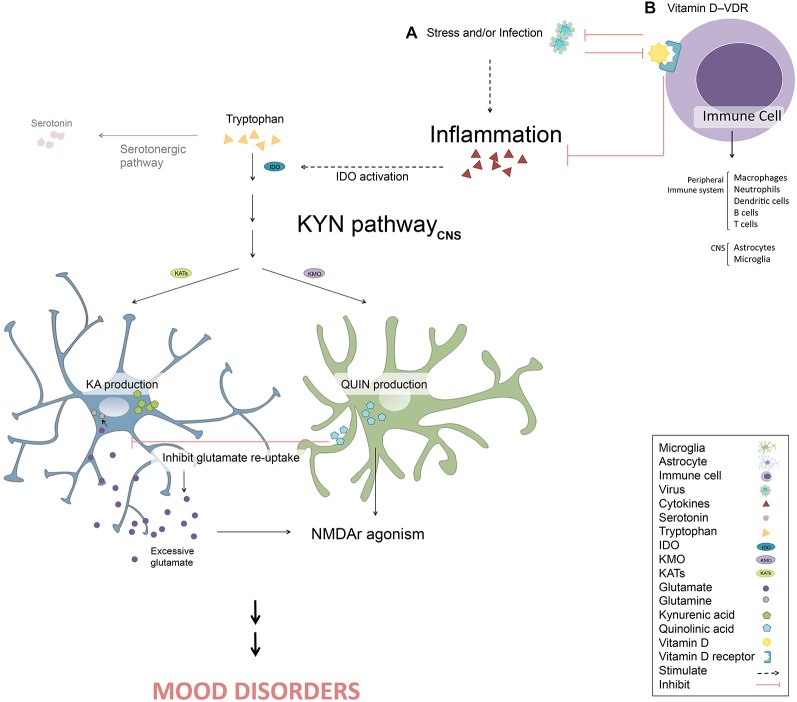
**Vitamin D and its receptor (VDR) in the cytokine-mediated onset of depression.** **(A)** Stress and/or infection activate the inflammatory response resulting in inflammatory cytokines production. These cytokines can trigger the kynurenine (KYN) pathway by stimulating indoleamine 2, 3-dioxygenase (IDO). Therefore, tryptophan enters the KYN pathway instead of the serotonergic pathway, resulting in the depletion of monoamines. Once the KYN pathway is triggered, kynurenic acid (KA) in astrocytes and quinolinic acid (QUIN) in microglia are synthesized. QUIN contributes to the activation of N-methyl-D-aspartate receptor (NMDAr) itself and by inhibiting glutamate re-uptake, resulting in excessive extracellular glutamate, inducing further NMDAr agonism. Excessive NMDAr activation leads to the disruption of the glutamatergic system, a key pathological feature in mood disorders. **(B)** The binding of vitamin D to VDR in a number of immune cells (e.g., macrophages, neutrophils, dendritic cells, B cells, T cells, astrocytes and microglia) leads to attenuation of the inflammatory response and stimulation of anti-microbial peptides production, potentially ameliorating symptoms of mood disorders. However, certain pathogens can also block or modulate epigenetic VDR activity.

Hence, the potential role of VDR influencing the peripheral and/or CNS immune response makes it an attractive target of study in the cytokine-mediated model of MDD (Figure 1). In addition, VDR susceptibility to epigenetic changes and likewise its role as epigenetic modulator raises the possibility of considering VDR as a potential biomarker in MDD. For instance, novel biomarkers, such as BDNF promoter methylation, are found to predict mood disorder patient’s response to treatment in some studies (D’Addario et al., 2012; Lopez et al., 2013; Tadić et al., 2014). Moreover, relations of VDR epigenetic modifications are already being widely investigated in cancer studies (Fetahu et al., 2014).

It is noteworthy that vitamin D and omega-3 fatty acids also take part in pathways other than the inflammatory network influencing the development of neuropsychiatric illnesses. It was proposed that deficiencies of vitamin D and omega-3 fatty acids in the brain affect serotonin-related mechanisms (Patrick and Ames, 2015). The heterodimer VDR-RXR stimulates serotonin synthesis by activating the tryptophan hydroxylase-2 transcription (Patrick and Ames, 2014). In addition, the omega-3 fatty acid eicosapentaenoic acid was suggested to facilitate serotonin release from neurons (Patrick and Ames, 2015). Moreover, omega-3 fatty acids were also found as a low-affinity ligand of VDR (Jurutka et al., 2007).

## Conclusions and Future Directions

Depression constitutes a distressing health concern topic. In the last decades, the importance of treating MDD has been accentuated, attracting public interest due to its increasing worldwide prevalence, particularly in high-income countries, threatening to become the leading cause of global disability. Moreover, currently available pharmacological antidepressants do not achieve desired results, producing severe side effects with high relapse rates. Increasing evidence suggests the involvement of the immune response. Cytokine-induced sickness behavior is proposed to culminate in MDD by promoting the activation of the KYN pathway and GR resistance, hence compromising the monoaminergic and glutamatergic neurotransmission along with the HPA axis hyperactivation. Therefore, new alternative options targeting the inflammatory response in mood disorders are being tested. Vitamin D appears to be a plausible candidate although current results from RCTs do not provide sufficient evidence to encourage its supplementation in MDD. It is, however, arguable that the intervention of vitamin D-VDR in different molecular mechanisms affecting the immune system is not fully understood. Research directed towards investigating the role of VDR in regulating the periphery and CNS immune response in relation to mood disorders is suggested. Moreover, another important issue raised in this review is the active participation of glial cells in the dysregulation of relevant mechanisms in the pathology of depression. Microglia and astrocytes are important CNS-immune mediators. However, interactions between VDR and glia have hardly been studied.

In view of the global importance of MDD and the gaps in knowledge that still exist concerning its pathophysiology, further pursuit of knowledge in regards to the glio-pathogenesis of the inflammatory system activation aiming to find better treatment options is highly warranted.

## Conflict of Interest Statement

The authors declare that the research was conducted in the absence of any commercial or financial relationships that could be construed as a potential conflict of interest.
